# Hospitalization due to varicella in the Netherlands

**DOI:** 10.1186/1471-2334-11-85

**Published:** 2011-04-05

**Authors:** Alies van Lier, Nicoline AT van der Maas, Gerwin D Rodenburg, Elisabeth AM Sanders, Hester E de Melker

**Affiliations:** 1 Department of Epidemiology and Surveillance, Centre for Infectious Disease Control, National Institute for Public Health and the Environment (RIVM), Postbox 1, 3720 BA Bilthoven, the Netherlands; 2 Department of Pediatric Immunology and Infectious diseases, University Medical Centre Utrecht (UMCU), Postbox 85090, 3508 AB Utrecht, the Netherlands

## Abstract

**Background:**

In the Netherlands, incidence of physician's consultations and hospitalizations for varicella is low compared to other countries. Better knowledge about the severity of varicella among Dutch hospitalized patients is needed. Therefore, a medical record research was conducted among hospitalized patients with diagnosis varicella.

**Methods:**

Hospital admissions due to varicella in 2003-2006 were obtained from the National Medical Register. Retrospectively, additional data were retrieved from the medical record of patients hospitalized with varicella in 23 Dutch hospitals using a standardized form. Analyses were performed using descriptive statistics.

**Results:**

The study population (N = 296) was representative for all varicella admissions in the Netherlands (N = 1,658) regarding age, sex, duration of admission and type of diagnosis. Complications were recorded in 76% of the patients (37% had at least one relatively severe complication). Bacterial super infections of skin lesions (28%), (imminent) dehydration (19%), febrile convulsions (7%), pneumonia (7%) and gastroenteritis (7%) were most frequently reported. No varicella-related death occurred within the study population and 3% of the patients had serious rest symptoms.

**Conclusions:**

It is not likely that the severity of varicella among hospitalized patients in the Netherlands differs from other countries. A considerable part of the varicella complications among hospitalized patients was rather moderate and can be treated effectively, although in a third of the hospitalized cases with complications, severe complications occurred. These data are relevant in the decision-making process regarding whether or not to introduce routine varicella vaccination in the Netherlands.

## Background

The varicella zoster virus (VZV) causes varicella (primary infection) as well as herpes zoster (reactivation of latent VZV in sensory nerve ganglia). Childhood varicella usually results in mild to moderate illness but serious complications like central nervous system involvement, pneumonia, secondary invasive bacterial infections and death may occur [1]. Since a life attenuated vaccine to prevent varicella has become available, many countries consider including it in their National Immunization Program. The United States was the first country that introduced universal childhood varicella vaccination. Their vaccination program started in 1995 (vaccine coverage among children aged 19 to 35 months increased nationally from 27% in 1997 to 89% in 2006) and has reduced overall disease incidence by 57% to 90%, hospitalizations by 75% to 88%, deaths by >74%, and direct inpatient and outpatient medical expenditures by 74% [2]. So far, in Europe varicella is listed as routine childhood vaccination in Germany and Greece. In some other European countries like Austria, Belgium, Cyprus, France, Italy, Poland, Spain, Sweden and Switzerland, routine childhood vaccination is only offered in some regions, only in the private sector or only to high risk groups and/or susceptible adolescents [3,4].

In the Netherlands [5], the number of varicella related consultations of a general practitioner (GP), hospital admissions and/or deaths per 100,000 inhabitants is low compared with other countries (pre-vaccine area), such as the United States [2], England and Wales [6-8] and Germany [9]. In England and Wales, where the health care system is most comparable to the Dutch situation, there were 507 GP-consults in the period 2001-2007 [6], 5.8 hospital admissions (based on data for England only) in 2000/2001-2008/2009 [7] and 0.038 deaths in 2000-2008 [8] per 100,000 inhabitants annually, which is roughly twice as much as the 238 GP-consults, 1.6 hospital admissions and 0.018 deaths per 100,000 inhabitants in the Netherlands in the period 2000-2008. It is not clear to what extent these differences can be ascribed to regional differences in the epidemiology of VZV, considering the fact that the mean age of infection in the Netherlands was lower than in England and Wales according to seroprevalence studies [10]. Other reasons might be differences in national surveillance systems, the health care system or health care seeking behavior. If these differences could be attributed to differences in the health care system or health care seeking behavior, which is in general more conservative in the Netherlands, there would be a possibility that Dutch hospitalizations due to varicella concern more severe cases than in other countries. Therefore, a medical record research was conducted among hospitalized patients with diagnosis varicella. This article presents the results of this study.

## Methods

Data on hospital admissions due to main and/or side diagnosis varicella (ICD-9 code 052) in the period 2003-2006 were obtained from the National Medical Register from research institute Prismant [11]. Clinical admissions were included only (admissions for one day or less were excluded). To get more detailed insight into the severity and complications of varicella among hospitalized patients, a medical record research was conducted. An acknowledged Medical Ethical Committee of the University Medical Centre Utrecht approved of the study that was in accordance with the European statements for good clinical practice, which includes the provisions of the Declaration of Helsinki of 1989. We invited 23 out of 91 Dutch hospitals (situation November 2009), including one of the eight academic hospitals, to participate in this study; five of these hospitals had more than 750 hospital beds in 2008. All the hospitals consented in participation and patients were sent an information letter asking permission to include their personal medical record data in the study. Patients that could not be reached because they had moved or died, patients that did not give (active) permission and patients whose medical record could not be traced were excluded from the study. For the remaining patients, trained and authorized medical students collected additional information on comorbidity, primary reason of admission, complications and rest symptoms of varicella out of the medical record using a standardized form, under supervision of a research physician. Afterwards, all complications were divided in relatively moderate or relatively severe complications by a medical doctor. For all deaths, including deceased patients that could not be included in the full research, additional information was collected on the cause of death and the time interval between the admission due to varicella and death. Varicella-related death was defined as death within one month after varicella related hospital admission, unless another cause of death than varicella was appointed.

### Statistical analysis

Differences between groups of patients were tested (two-tailed) by using Fisher's exact test (sex, type of hospital, type of diagnosis, having a complication), Pearsons χ^2 ^test (ICD-9 code), Median Test (median age, median duration of admission) or a t-test (number of complications). For all tests, a p-value of <0.05 was considered to be statistically significant.

## Results

### Patient characteristics

**Figure 1:**
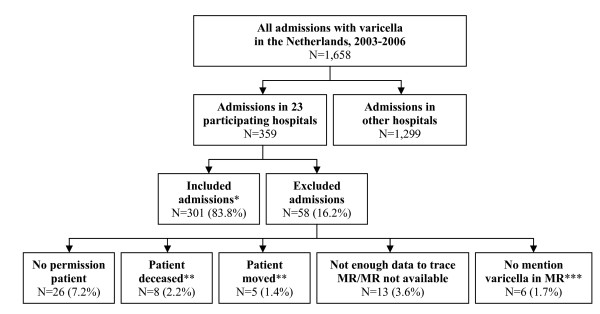
**Flow chart of in- and excluded admissions in the medical record research**. MR = medical record. * The included admissions concerned 296 individual patients (some patients were registered more than once) ** Not included because it was not possible to inform these patients (or their relatives) *** Included at first but excluded during the study because there was no mention in MR of a varicella episode (or only mention of a suspicion that was withdrawn after further investigation).

In the 23 hospitals that consented in participation, 359 admissions with main and/or side diagnosis varicella were registered in the National Medical Register in the period 2003-2006. This covered 21.7% of all the national admissions with main and/or side diagnosis varicella in the Netherlands in this period. The medical record research could be conducted for 301 admissions (83.8%, see Figure 1); this concerned 296 individual patients (some patients were registered more than once). There was no significant difference in median age, sex, median duration of admission, type of diagnoses (main or side) and ICD-9 codes between the study population and all Dutch hospitalized patients in the National Medical Register in 2003-2006 (Table 1). When comparing the type of hospital, the proportion admitted to an academic hospital was lower for the study population (5.1%) compared to the total group of hospitalized patients (11.2%).

**Table 1:** Background characteristics of study population medical record research versus all hospitalized patients with main or side diagnosis varicella in 2003-2006

		Study population		All patients 2003-2006		
		
		N	%	N	%	p-value
Age	0 year	94	31.8%	492	29.7%	
	1-4 year	145	49.0%	760	45.8%	
	≥5 year	57	19.3%	406	24.5%	
*Median*		*1*	*years*	*2*	*years*	*p = 0.103*

Sex	Male	179	60.5%	931	56.2%	p = 0.181
	Female	117	39.5%	727	43.8%	

Duration of admission	<1 day	45	15.3%	239	14.4%	
	1-4 days	164	55.6%	875	52.8%	
	≥5 days	86	29.2%	543	32.8%	
*Median*		*3.6*	*days*	*3.3*	*days*	*p = 0.658*

Type of hospital	Academic	15	5.1%	185	11.2%	p = 0.001
	Other	281	94.9%	1473	88.8%	

Type of diagnosis	Main	190	64.2%	1100	66.3%	p = 0.465
	Side	106	35.8%	558	33.7%	

ICD-9 code*	052.0	2	1.1%	21	1.9%	p = 0.436
main diagnosis	052.1	11	5.8%	82	7.5%	
	052.7	39	20.5%	261	23.7%	
	052.8	16	8.4%	66	6.0%	
	052.9	122	64.2%	670	60.9%	

ICD-9 code*	052.0	0	0.0%	1	0.2%	p = 0.489
side diagnosis	052.1	0	0.0%	7	1.3%	
	052.7	12	11.3%	72	12.9%	
	052.8	1	0.9%	17	3.0%	
	052.9	93	87.7%	461	82.6%	

Total		296	100.0%	1658	100.0%	

* ICD-9 code: 052.0 = postvaricella encephalitis, 052.1 = varicella (hemorrhagic) pneumonitis, 052.7 = varicella with other specified complications, 052.8 = varicella with unspecified complication, 052.9 = varicella without complication

The study showed that 114 of 296 patients (39%) had an underlying chronic condition that is known from the literature to increase the chance of getting severe varicella or complications due to varicella. Most frequently reported pre existent conditions were lung disorders, skin disorders (mainly eczema or constitutional eczema), malignancy/leukemia, premature birth and immunosuppressive therapy (Table 2). Among 'other chronic condition' various conditions were mentioned, such as epilepsy, Down syndrome and a history of febrile convulsions. Patients with an underlying chronic condition were somewhat older than patients without such a condition (median age 2 versus 1 years, p = 0.005) and had a longer duration of admission (median duration 4.1 versus 2.9 days, p = 0.005). There were no significant differences in the occurrence of at least 1 complication (71% versus 78%, p = 0.164) and mean number of complications (1.0 versus 1.1, p = 0.446) between hospitalized patients with and without an underlying chronic condition.

**Table 2:** Reported underlying chronic conditions in medical record research

Condition	N	%
No underlying chronic condition	176	60.7%
Underlying condition	114	39.3%
- Lung disorder	33	
- Skin disorder	27	
*Eczema*	*(18)*	
*Constitutional eczema*	*(7)*	
- Malignancy/leukemia	18	
- Premature birth	13	
- Immunosuppressive therapy	11	
- Cardiovascular disease	6	
- Primary or congenital immune disorder	4	
- Thalassaemia	3	
- Disorder of the thyroid gland	3	
- Organ or bone marrow transplantation	2	
- HIV	2	
- Nephrotic syndrome	2	
- Diabetes mellitus	2	
- Liver disease	1	
- Other chronic condition	27	
*Epilepsy*	*(5)*	
*Down syndrome*	*(4)*	
*Febrile convulsions*	*(2)*	
Unknown	6	-

Total	296	100%

### Disease course and outcome

For 225 patients (76%) one or more varicella-related complications were reported in the medical record (72% among 0 year olds, 86% among 1-4 year olds and 58% among patients aged 5 year or older). Within the group of patients with complication(s), 37% had an underlying pre existent condition (30% among 0 year olds, 39% among 1-4 year olds and 44% among patients aged 5 year or older). Of all 322 complications mentioned, 95 (30%) were considered to be relatively severe complications (see Table 3 for the division in relatively moderate and severe complications). Of the 225 patients with a complication, 83 (37%) had at least one relatively severe complication. Most frequently reported complications were bacterial super infections of skin lesions (28%), (imminent) dehydration (19%), febrile convulsion (7%), pneumonia (7%) and gastroenteritis (7%) (Table 3). Based on the median duration of admission, the five most frequently reported complications can be ordered as follows: pneumonia (4.7 days), gastroenteritis (4.6 days), bacterial super infections of skin lesions (3.7 days), (imminent) dehydration (2.8 days) and febrile convulsion (1.6 days).

**Table 3:** Reported complications in medical record research

Complication	N	%	Complication	N	%
**Neurological**			**Gastrointestinal tract**		
Cerebellitis, ataxia^2^	9	3.0%	Gastroenteritis^1^	21	7.1%
Febrile convulsion^2^	22	7.4%	Stomatitis^1^	13	4.4%
Convulsion without fever^2^	1	0.3%	(Imminent) dehydration^1^	57	19.3%
Meningitis/encephalitis^2^	7	2.4%	Liver disorder^2^	2	0.7%
Cerebral vasculitis/infart/bleeding^2^	1	0.3%	**Secondary bacterial infections**		
**Lower respiratory tract**			Sepsis^2^	6	2.0%
Pneumonia^2^	22	7.4%	Osteomyelitis^2^	1	0.3%
Bronchitis^1 ^or bronchiolitis^2^	6	2.0%	Bacterial arthritis^2^	4	1.4%
**Upper respiratory tract, ENT and eye**			**Haematological/coagulation disorders**		
Otitis media^1^	16	5.4%	Thrombocytopenia^2^	5	1.7%
Faryngitis/tonsillitis^1^	10	3.4%	Coagulation disorder^2^	3	1.0%
Conjunctivitis^1^	7	2.4%	Leuco- and/or erythrocytopenia/anaemia^1^	8	2.7%
**Skin/subcutis**			**Other complications**		
Bacterial super infections of skin lesions^1^	82	27.7%	Reactive arthritis^2^	1	0.3%
Staphylococcal scalded skin syndrome^2^	1	0.3%	Other^1/2^	15	5.1%
Erythema multiforme exsudativum^1^	1	0.3%			
Necrotizing fasciitis^2^	1	0.3%			

^1 ^= relatively moderate complication, ^2 ^= relatively severe complication

For 36 patients (12%) rest symptoms were reported at discharge. In 10 of 296 patients (3%) this concerned serious rest symptoms like residual ataxia or coordination disorder (N = 8) or cerebral nerve paralysis (N = 2). In another 14 patients (5%) mild rest symptoms were reported, for example bad healing lesions (N = 10) or scars (N = 4). There were no deaths considered to be varicella related, although for one deceased person that could not be included in the study we could not totally exclude the possibility.

### Comparison between National Medical Register and medical record research

The attribution to main or side diagnosis in the National Medical Register corresponded to the medical record for 71% of the patients (Table 4). For 3% of the patients varicella was found not to be the primary reason for admission while this concerned a main diagnosis according to the National Medical Register. For 26% of the patients varicella was retrospectively considered to be the primary cause of admission while it was only registered as side diagnosis in the National Medical Register. In these 77 cases, the main diagnosis as registered in the National Medical Register often seemed a complication related to varicella (N = 66) or sometimes a chronic condition associated with a higher risk on a severe course of varicella (N = 5), like for instance leukemia. The most frequently mentioned main diagnoses were bacterial super infections of skin lesions (26%), febrile convulsion (17%) and (imminent) dehydration (12%).

**Table 4:** Comparison of National Medical Register with medical record research with regard to the attribution of main or side diagnosis varicella

	National Medical Register		
	
Medical record research	Main diagnosis	Side diagnosis	Total
Admission primary due to varicella	180 (61%)	77 (26%)	257
Admission not primary due to varicella	9 (3%)	29 (10%)	38
Total	189	106	295

Moreover, almost half (45%) of the varicella complications as retrieved from the medical record was not registered as such in the National Medical Register. A detailed comparison between the medical record research and the National Medical Register regarding varicella complications is presented in Table 5. For example, only 50 of the 82 (61%) bacterial super infections of skin lesions were coded in the National Medical Register. More important, a number of serious complications such as cerebellitis/ataxia, meningitis/encephalitis and sepsis were only partially registered in the National Medical Register. Of note is that the specific ICD-codes for postvaricella encephalitis and varicella (haemorrhagic) pneumonitis were not used in the National Medical Register for all patients who suffered from meningitis/encephalitis or pneumonia due to varicella according to the medical record.

**Table 5:** Comparison of National Medical Register (ICD-9 codes*) and medical record research with regard to registered complications

Complications	National Medical Register (LMR)						
	
Medical record research	052.0 N	052.1 N	052.7 N	052.8 N	052.9 N	Total N	Complication registered N (%)
**Neurological**							
Cerebellitis, ataxia	2	-	6	-	1	9	4 (44.4%)
Febrile convulsion	-	-	2	2	18	22	17 (77.3%)
Convulsion without fever	-	-	-	-	1	1	1 (100.0%)
Meningitis/encephalitis	-	-	1	1	5	7	3 (42.9%)
Cerebral vasculitis/infart/bleeding	-	-	1	-	-	1	1 (100.0%)
**Lower respiratory tract**							
Pneumonia	-	9	2	2	9	22	17 (77.3%)
Bronchitis or bronchiolitis	-	-	1	1	4	6	^a ^4 (66.7%)
**Upper respiratory tract, ENT and eye**							
Otitis media	-	-	4	1	11	16	6 (37.5%)
Faryngitis/tonsillitis	-	-	1	-	9	10	5 (50.0%)
Conjunctivitis	-	-	3	-	4	7	1 (14.3%)
**Skin/subcutis**							
Bacterial super infections of skin lesions	-	-	28	9	45	82	^b ^50 (61.0%)
Staphylococcal scalded skin syndrome	-	-	-	-	1	1	1 (100.0%)
Erythema multiforme exsudativum	-	-	-	-	1	1	0 (0.0%)
Necrotizing fasciitis	-	-	-	1	-	1	0 (0.0%)
**Gastrointestinal tract**							
Gastroenteritis	-	2	5	1	13	21	12 (57.1%)
Stomatitis	-	-	2	1	10	13	^c ^9 (69.2%)
(Imminent) dehydration	-	-	8	4	45	57	26 (45.6%)
Liver disorder	-	1	-	1	-	2	1 (50.0%)
**Secondary bacterial infections**							
Sepsis	-	-	2	1	3	6	1 (16.7%)
Osteomyelitis	-	-	1	-	-	1	1 (100.0%)
Bacterial arthritis	-	-	2	-	2	4	3 (75.0%)
**Haematological/coagulation disorders**							
Thrombocytopenia	-	1	-	1	3	5	1 (20.0%)
Coagulation disorder	-	-	-	2	1	3	1 (33.3%)
Leuco- and/or erythrocytopenia/anaemia	-	1	2	2	3	8	2 (25.0%)
**Other complications**							
Reactive arthritis	-	-	-	-	1	1	1 (100.0%)
Other	-	1	4	-	10	15	10 (66.7%)
**Total**	**2**	**11**	**51**	**17**	**215**	**296**	

* ICD-9 code: 052.0 = postvaricella encephalitis, 052.1 = varicella (hemorrhagic) pneumonitis, 052.7 = varicella with other specified complications, 052.8 = varicella with unspecified complication, 052.9 = varicella without complication

^a ^acute upper respiratory infections registered in LMR were also included

^b ^all bacterial infections (also when it was not clear that it concerned the skin) registered in LMR were included

^c ^volume depletion disorder and feeding difficulties and mismanagement, registered in LMR were also included (except for patients for whom (imminent) dehydration was mentioned by the medical student as well)

## Discussion

The medical record research showed that varicella complications occurred in the majority (76%) of hospitalized patients; 37% of these patients had at least one relatively severe complication. The most frequently mentioned complication that came forward, bacterial super infections of skin lesions, usually can be treated effectively by antibiotics. (Imminent) dehydration, the second most frequently mentioned complication, can normally be treated effectively by administering a rehydration solution. As expected due to the small study size, we did not find some unusual complications of varicella known from the literature such as Reye's syndrome, toxic shock syndrome, neonatal varicella and proliferative glomerulonephritis. Remarkably, there were no differences in the occurrence of complications between hospitalized patients with and patients without an underlying chronic condition. In 3% of the patients in this study serious rest symptoms were reported. Based on the cause of death and/or time between admission and death, none of the deaths were considered to be varicella related. This also counts for 7 of the 8 deceased patients that could not be included in the study. For one deceased patient for whom both date and cause of death were unknown, we cannot totally exclude the possibility that death was varicella related. However, this patient was admitted because of moderate complications (infected skin lesions and dehydration) that are unlikely reasons for case fatality. At this moment we have therefore no reason to assume that the lower number of admitted varicella patients in the Netherlands concerned more severe or other presenting cases than in other countries.

It is not easy to make a comparison of the severity of varicella complications among hospitalized patients between different countries. In a German study [9], where the incidence of varicella-related hospital admissions was estimated to be approximately three times higher than in the Netherlands (Germany 19.7 per 100,000 in children ≤16 years of age, the Netherlands 6.8 per 100,000 children ≤15 years of age), the proportion of certain complications among hospitalized varicella cases was rather comparable with the proportions found in our study. In this German study they found a febrile convulsion in 7.6% (Netherlands 8.7% in the same age-group ≤16 years of age), meningo-encephalitis in 5.7% (Netherlands 1.6%), cerebral vasculitis/infarct in 0.7% (Netherlands 0.4%), staphylococcal scalded skin syndrome in 0.7% (Netherlands 0.4%), coagulation disorder in 5.6% (Netherlands 2.8%) and sepsis in 2.3% (Netherlands 2.0%) of the hospitalized varicella patients. The reason behind the difference between both countries in the proportion of patients with meningo-encephalitis and coagulation disorder to a lesser extent is not clear. A possible explanation could be the difference in median age of the study population (for Germany 3.3 years and for the Netherlands 1 year) because the severity of varicella complications is known to be related to increasing age. Another possibility could be the differences in data collection methods between the German study (nationwide, prospective, hospitals were asked each month to send a reporting card to report all patients with a varicella related condition, including postinfectious varicella complications, and for these patients additional information was collected) and the Dutch study (not nationwide, retrospective, based on available information in the medical record of patients).

A limitation of this study is that the selection of patients was based on registration of varicella (as main or side diagnosis) in the National Medical Register. In a considerable part of patients, varicella was incorrectly registered as side diagnosis instead of main diagnosis. Furthermore, a considerable part of the patients were incorrectly registered in the National Medical Register as 'varicella without complication' or as 'varicella with unspecified complication', sometimes despite the fact that additional codes were registered in the National Medical Register that were very likely complications caused by varicella. We cannot exclude the possibility that varicella might not have been registered at all in another unknown number of patients. This means that there is a chance of underestimation of the number of hospitalizations due to varicella. Future research should therefore focus on following up patients that consult their GP due to varicella, to see how often they are referred to a hospital.

Only a part of all Dutch hospitals participated in the study, representative for 21.7% of all the admissions with varicella in the Netherlands in 2003-2006, which limits our study sample size. Furthermore, the records of 3% of all patients with varicella diagnosis could not be included due to lack of permission or missing records in the hospital. However, the study population was representative for all hospitalized patients with varicella diagnosis in the Netherlands in 2003-2006, with the exception of the type of hospital, but within the study population both median duration of admission and the proportion with at least one complication in the academic hospital did not differ significantly from other hospitals.

## Conclusions

This study showed that a considerable part of the varicella complications among hospitalized cases was rather moderate and can normally be treated effectively, although in a third of the hospitalized cases with complications, severe complications occurred. Furthermore, in spite of the lower incidence of hospital admissions due to varicella, there is no reason to assume that the severity of varicella among hospitalized patients in the Netherlands differs from other countries. These data are relevant in the decision-making process regarding whether or not to introduce routine varicella vaccination in the Netherlands.

## Competing interests

The authors declare that they have no competing interests.

## Authors' contributions

AvL participated in the design and coordination of the study and drafted the manuscript, NvdM and GR participated in the design and coordination of the study and critically revised the manuscript, ES and HdM participated in the design of the study and critically revised the manuscript. All authors read and approved the final manuscript.

## Pre-publication history

The pre-publication history for this paper can be accessed here:

http://www.biomedcentral.com/1471-2334/11/85/prepub
